# Lung Sarcoidosis in Etanercept Treated Rheumatoid Arthritis Patient: A Case Report and Review of the Literature

**DOI:** 10.1155/2014/358567

**Published:** 2014-07-03

**Authors:** Supat Thongpooswan, Adriana Abrudescu

**Affiliations:** Mount Sinai School of Medicine, Queens Hospital Center, Jamaica, NY 11432, USA

## Abstract

We report a 55-year-old female with seropositive rheumatoid arthritis for 10 years who developed large mediastinal and hilar adenopathy while receiving etanercept therapy. Chest high resolution computed tomography (HRCT) showed mediastinal lymph nodes with size of 2.3 × 3.1 centimeters. Right paratracheal lymph node biopsy showed nonnecrotizing epithelioid granulomata. All infectious studies of pulmonary lymph node tissues were negative. Etanercept was discontinued. Follow-up HRCT 6 months later showed resolution of mediastinal lymph nodes. This report should increase awareness of pulmonary sarcoidosis development in patient treated with tumor necrosis factor-alpha blocking agent, etanercept.

## 1. Introduction

A soluble tumor necrosis factor (TNF) receptor fusion protein, etanercept (Enbrel), has been used widely around the world for treatment of rheumatoid arthritis (RA) for many years. Various precautions and side effects have been reported which were mainly infection and malignancy. However in the past few years, there are small number of literatures possible that involve TNF therapy, mainly etanercept, in the development of pulmonary aseptic granulomatosis such as sarcoidosis. Our case report supports possible relationship between etanercept therapy and the development of sarcoid-like granulomatosis.

## 2. Case Presentation

**Figure 1:**
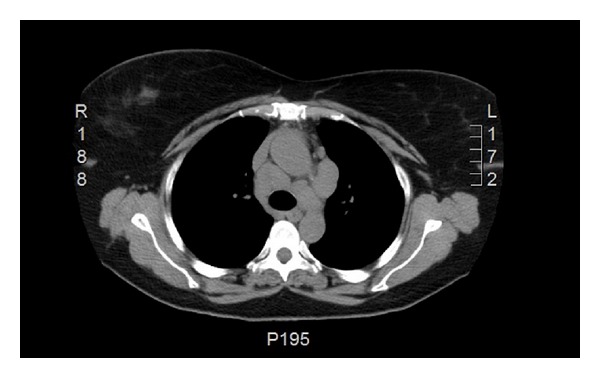
HRCT of the thorax demonstrating mediastinal lymph nodes and the largest was 2.3 × 3.1 centimeters.

**Figure 2:**
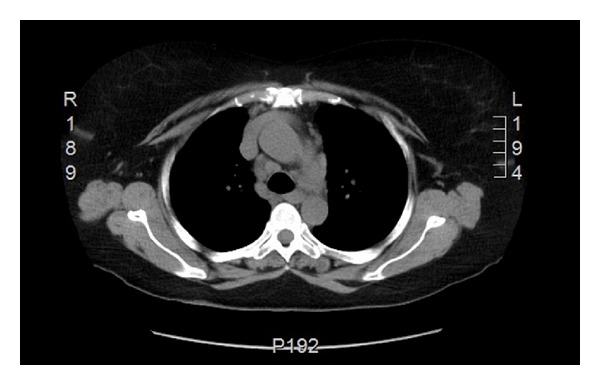
Follow-up thorax CT demonstrating resolution of mediastinal lymph nodes.

**Figure 3:**
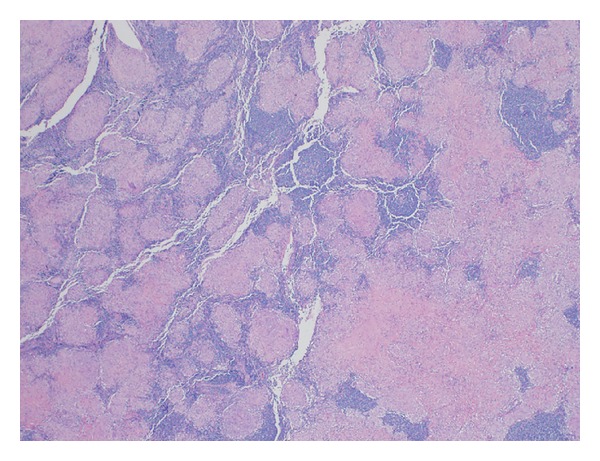
Right paratracheal lymph node histology; low magnification.

**Figure 4:**
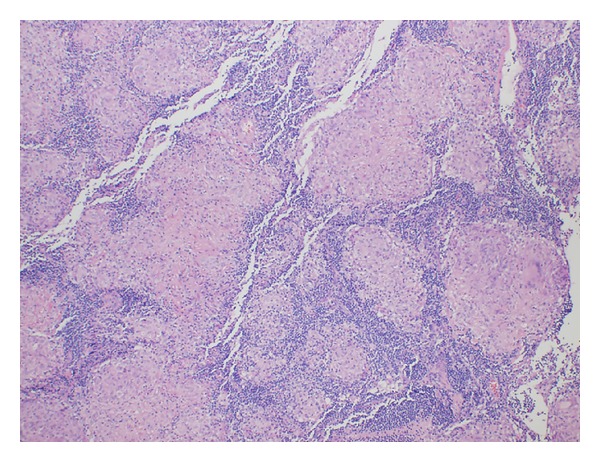
Right paratracheal lymph node histology; medium magnification.

We report a case of 55-year-old Guyanese female with the history of seropositive rheumatoid arthritis (RA) for 10 years and history of latent tuberculosis. Isoniazid treatment was not completed due to abnormal liver function tests and rifampin was completed at 4 months. Her RA was poorly responsive to methotrexate 20 mg weekly, hydroxychloroquine 400 mg daily, and chronic prednisone therapy between 10 and 20 mg daily. She had been tried on leflunomide to control her symptoms but experienced severe hair loss. She was started on adalimumab (Humira), but with no clinical improvement, and also she developed rash 7 years ago. Weekly subcutaneous etanercept 50 mg was started after adalimumab with good disease control for 3 years in the absence of pulmonary or mediastinal abnormality on chest computed tomography; however it was stopped due to insurance issue 3 years ago. She started to develop cough and dyspnea. The blood analysis demonstrated ESR 40 mm/h, CRP 1.35 mg/dL, negative antinuclear antibody, hemoglobin 12.5 g/dL, white blood cell 10.3 K/mcl, platelet 296 K/mcl, and normal renal and hepatic functions. High resolution computed tomography (HRCT) showed ground glass opacities on bilateral lower lungs. Echocardiography was normal. Methotrexate was stopped. Her cough and shortness of breath improved. Bronchoscopy revealed no endobronchial lesion. The microbiological analysis of bronchial lavage, bacterial, fungal, and mycobacterial cultures, and pneumocystis smear was negative. Cytologic analysis was negative for malignant, reactive bronchial epithelial, pneumocytes, and lymphocytes cell. Transbronchial biopsy showed focal emphysematous changes, mild interstitial pneumonitis. Due to active RA, prednisone was increased to 10 mg twice daily and weekly subcutaneous etanercept 50 mg was started again. Her RA symptoms including synovitis subsequently improved and prednisone was able to slowly taper down to 5 mg every other day. After taking etanercept for 12 months, follow-up HRCT showed decreasing bilateral ground glass opacities. However mediastinal lymph nodes were increasing in size to 2.3 × 3.1 centimeters (Figure 1). Mediastinoscopy was done and showed enlarged right anterior lymph nodes. Tissue biopsy showed nonnecrotizing epithelioid granulomata (Figures 3 and 4). All infectious studies of lymph node tissues were negative. Etanercept was discontinued. Follow-up chest CT 12 months later showed resolution of mediastinal lymph nodes (Figure 2). The diagnosis of etanercept induced pulmonary sarcoidosis was made.

## 3. Discussion

In the United States, three biological agents have been widely used for RA treatments: etanercept (Enbrel) which is a soluble TNF receptor fusion protein, infliximab (Remicade) which is a chimeric monoclonal antibody, and adalimumab (Humira) which is a human monoclonal antibody [1]. These three agents have been proven effective in RA treatment. Serious side effects, mainly, infection and malignancy, were described, rare for granulomatous disease like pulmonary sarcoidosis [2, 3].

The formation of granuloma begins with antigen-driven immune process that is mediated by activated macrophages and T-helper 1 CD 4 cells associated with increased production of several cytokines including interleukin- (IL-) 12, IL-18, and TNF [4]. It seems reasonable that TNF inhibitor would be useful for granulomatous disease, like sarcoidosis [5]. Infliximab has been shown in preliminary randomized controlled trial (RCT) with significant improvement in % predicted FVC in severe, chronic, symptomatic sarcoidosis [6].

However, etanercept, a soluble TNF receptor fusion protein, was not found to be effective in RCT of refractory ocular sarcoidosis treatment and pulmonary sarcoidosis treatment trial [7, 8].

Paradoxically, cases have been reported on the development of sarcoidosis in RA during etanercept treatment [9–14]. The incidence of this adverse effect has been reported to be occurring at 1/2800 [12]. The majority were women, with mean age of 49. The TNF antagonist most often used was etanercept followed by infliximab and adalimumab [9]. Hilar and mediastinal adenopathy were the most common radiological finding [9, 15]. Most of the time, mediastinal or pulmonary symptoms resolved after withdrawal of etanercept, which is consistent with our case [13].

The pathogenesis is still unclear. Firstly, etanercept may not inhibit other cytokines activation such as IL-2 and IL-18 which can support granuloma formation [9]. Secondly, etanercept partially blocks TNF, leading to contribution in granuloma formation [16]. Thirdly, granuloma may be secondary to late adverse complication of adalimumab since 7 years which have been reported in case series [12]. However the median delay between anti-TNF drug onset and diagnosis of sarcoid-like granulomatosis was 11 (1–21) months for adalimumab which was different from our case.

There is an unusual aspect of this case that we cannot scientifically explain why she did not develop pulmonary sarcoidosis with the first round of etanercept. In fact, the length of therapy of the first round was longer than the second round. This may be the same mechanism that explains why a different patient has different mean onset duration of pulmonary sarcoidosis. Further studies or large case series would help better understand this mechanism.

However, in our case, her mediastinal and hilar adenopathy resolved after etanercept withdrawal thus suggesting association of pulmonary sarcoidosis and etanercept treatment.

## 4. Conclusion

We describe a RA case that developed pulmonary sarcoidosis while being treated with etanercept. The mechanism of pulmonary sarcoidosis is still unclear. It is important for the physician to be aware of this uncommon side effect. Our case is different from others because our patient develops sarcoid-like granulomatosis during the second round of etanercept therapy. This case will emphasize the importance of repeated chest imaging when respiratory symptoms developed. This case report will support further studies to clarify TNF inhibitor induced sarcoidosis.
